# Structural Characterization of the SMRT Corepressor Interacting with Histone Deacetylase 7

**DOI:** 10.1038/s41598-017-03718-5

**Published:** 2017-06-16

**Authors:** Danielle C. Desravines, Itziar Serna Martin, Robert Schneider, Philippe J. Mas, Nataliia Aleksandrova, Malene Ringkjøbing Jensen, Martin Blackledge, Darren J. Hart

**Affiliations:** 1 European Molecular Biology Laboratory, Grenoble Outstation, 71 Avenue des Martyrs, CS90181, 38042 Grenoble Cedex 9, France; 2 Unit of Virus Host-Cell Interactions (UVHCI), University Grenoble Alpes, CNRS, EMBL, 38042 Grenoble, France; 3 Institut de Biologie Structurale (IBS), CEA, CNRS, University Grenoble Alpes, 38044 Grenoble, France; 4 Dunn School of Pathology, University of Oxford, South Parks Road, OX13 RE Oxford, UK; 5 University Lille, CNRS, UMR 8576 - UGSF - Unité de Glycobiologie Structurale et Fonctionnelle, 59000 Lille, France

## Abstract

The 2525 amino acid SMRT corepressor is an intrinsically disordered hub protein responsible for binding and coordinating the activities of multiple transcription factors and chromatin modifying enzymes. Here we have studied its interaction with HDAC7, a class IIa deacetylase that interacts with the corepressor complex together with the highly active class I deacetylase HDAC3. The binding site of class IIa deacetylases was previously mapped to an approximate 500 amino acid region of SMRT, with recent implication of short glycine-serine-isoleucine (GSI) containing motifs. In order to characterize the interaction in detail, we applied a random library screening approach within this region and obtained a range of stable, soluble SMRT fragments. In agreement with an absence of predicted structural domains, these were characterized as intrinsically disordered by NMR spectroscopy. We identified one of them, comprising residues 1255–1452, as interacting with HDAC7 with micromolar affinity. The binding site was mapped in detail by NMR and confirmed by truncation and alanine mutagenesis. Complementing this with mutational analysis of HDAC7, we show that HDAC7, via its surface zinc ion binding site, binds to a 28 residue stretch in SMRT comprising a GSI motif followed by an alpha helix.

## Introduction

**Figure 1:**
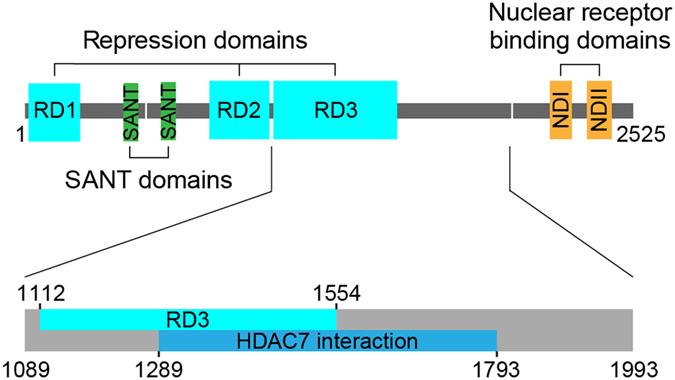
Functional and structural domains of the SMRT protein. Top, general schematic of the entire protein; bottom, close-up view of the region investigated in this work.

The major role of histone deacetylases (HDACs) is to deacetylate lysines of different protein substrates, commonly histones, where they lead to compaction of the chromatin structure and gene repression. The human HDAC family comprises 18 members divided into 4 classes based on sequence homology. Among these are class I HDACs 1, 2, 3 and 8 with a catalytic domain of 377–488 amino acids^1, 2^, and class II HDACs that are 855 to 1122 amino acids long and contain an additional N-terminal regulatory domain^1–3^. Class II is further subdivided into IIa (HDAC4, 5, 7 and 9) and IIb (HDAC6 and 10)^1, 4^. Crystal structures of class I and II catalytic domains reveal a common active site with a deep pocket containing a conserved zinc-binding site. HDAC7, first identified as a component of a multiprotein complex^5^, has a similar catalytic domain structure to other HDACs^6–8^. As with other class IIa enzymes, the mechanistically important tyrosine (Tyr306) is replaced by a histidine, resulting in a barely detectable or “cryptic” enzymatic activity^6, 7, 9^. This inactivation of an otherwise conserved active site suggests that class IIa enzymes may instead function as acetyl-lysine readers similar to bromodomains^10^, with deacetylation activity being contributed by class I HDACs in the context of a corepressor complex. HDAC7 functionality is dependent on the presence of HDAC3, and both enzymes were shown to associate with SMRT and N-CoR1 corepressors^11^. SMRT (silencing mediator of retinoid acid and thyroid hormone receptor), also known as N-CoR2, is a 2525 amino acid protein identified as binding to unliganded nuclear receptors^12^ (Fig. 1). It has 40% sequence identity to N-CoR1; however, the two proteins are non-redundant and knockout of either of them results in embryonic death in mice^13, 14^. Deregulation of corepressor function via defective protein-SMRT interactions has been implicated in many cancers^15^ including acute promyelocytic^16, 17^, acute myeloid^18^ and common acute lymphoblastic leukemias^19^, as well as other conditions, e.g. resistance to thyroid hormone (RTH) syndrome^20^.

Despite its large size, only two small structured SANT-like domains have been characterized in SMRT. One is the deacetylase activating domain (DAD) that recruits and activates HDAC3 in a inositol phosphate-mediated fashion^21, 22^, the other forms a histone interaction domain^23^. HDAC7 has been shown to bind within a 500 amino acid region of SMRT (residues 1289–1793)^24^ via a second zinc ion on the surface of the catalytic domain^7^. Analysis of the SMRT sequence using computational predictors suggests that most of its chain is unstructured (Fig. S1) and that, consequently, it belongs to the class of intrinsically disordered proteins (IDPs) that are functional despite a lack of stable three-dimensional structure. Research over the last two decades has underlined the important roles of IDPs in biology, notably in processes such as signaling, regulation, or mediation of protein-protein interactions^25–31^. SMRT conforms well to this profile, forming interactions with a wide range of proteins, including HDACs, nuclear receptors and transcription factors^18, 32–34^.

Development of class IIa HDAC inhibitors (HDIs) is complicated by the very low catalytic activity of these enzymes and a poor understanding of their function^35^. However, class IIa HDACs have a number of roles in development and physiology^36^ and are implicated in cancer^15, 37, 38^, making them attractive pharmacological targets. Most inhibition strategies target the HDAC active site with metal-binding compounds (e.g. trichostatin A or suberanilohydroxamic acid). Generally, HDIs lack specificity due to cross-reactivity with other HDACs and metalloenzymes^39^, although there has been some progress towards class IIa-specific HDIs^39^.

An alternative strategy to inhibit class IIa HDACs would be to block their recruitment into the SMRT corepressor complex, which might avoid problems of pan-HDAC or metalloenzyme binding. Towards this goal, we screened a library of SMRT constructs to obtain soluble fragments, then used NMR spectroscopy and biophysical methods to localize and characterize the HDAC7-SMRT binding interface. NMR is well suited for studying intrinsically disordered proteins^40^ and allowed us to characterize both structural propensities and the HDAC7 binding site of SMRT with single-residue precision. Our data lay the foundation for the development of selective inhibitors of corepressor assembly.

## Results

### Expression of soluble fragments of SMRT

The region of SMRT interacting with class IIa HDACs was previously localized to the region of repression domain (RD) 3 using yeast two-hybrid deletion analysis^11, 41, 42^ and more specifically to residues 1289–1793^24^ (Fig. 1). We synthesized an *E*. *coli* codon-optimized SMRT gene fragment (encoding residues 1089–1993) and screened it for soluble constructs using the ESPRIT (expression of soluble proteins by random incremental truncation) method^43, 44^. Initially we obtained 51 constructs yielding soluble purifiable protein visible by western blot. Most fall in the size range of 100 to 200 amino acids and, collectively, cover most of the SMRT fragment investigated. Based on yield from small-scale expression trials, proteolytic stability and sequence position, six constructs (1122–1254, 1255–1452, 1276–1405, 1784–1993, 1798–1966 and 1882–1993) were selected for isotope-labeled expression, purification and NMR spectroscopy. In agreement with bioinformatic predictions (e.g. IUPRED^45^), resistance to aggregation at 80 °C and abnormal migration of proteins on SDS-PAGE gels, the reduced dispersion of signals in the ^1^H dimension of ^15^N-^1^H heteronuclear single quantum coherence (HSQC) NMR spectra confirmed the protein fragments as disordered (Supplementary Fig. S2).

### Identification and characterization of HDAC7-interacting SMRT fragments

**Figure 2:**
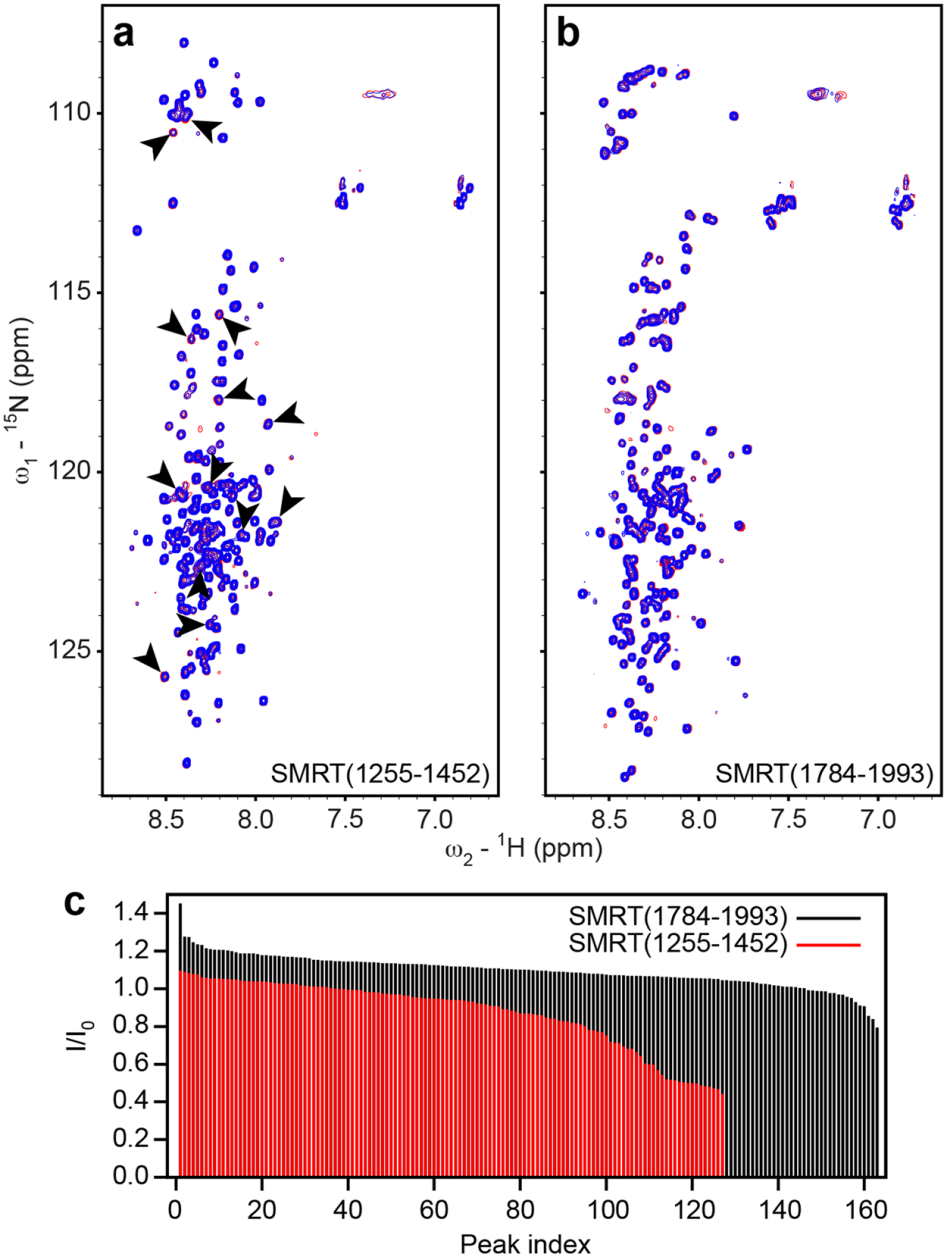
Interaction of SMRT fragments with HDAC7. (**a**,**b**) Superposition of ^15^N-^1^H HSQC spectra of SMRT fragments 1255–1452 (**a**) and 1784–1993 (**b**) in the absence (red) and presence (blue) of equimolar amounts of unlabeled HDAC7. In (**a**), selected peaks exhibiting reduced intensity in the presence of HDAC7 are indicated by arrows. See also Supplementary Fig. S4. (**c**) Relative peak intensity ratios I/I_0_ between spectra with and without added HDAC7 of SMRT(1255–1452) (red) and SMRT(1784–1993) (black), showing significant signal attenuation only for SMRT(1255–1452). Since sequential resonance assignment was not performed for SMRT(1784–1993), peaks are arbitrarily numbered and sorted for relative intensity ratio for both SMRT fragments to facilitate comparison. Only non-overlapping peaks were considered. Additional peaks for the SMRT(1784–1993) construct result from the presence of a C-terminal biotin tag.

For interaction studies, equimolar mixtures of ^15^N-labeled SMRT constructs and unlabeled HDAC7 protein were prepared. SMRT (1255–1452) and SMRT (1276–1405) interact with HDAC7, as apparent from reduced or vanishing spectral peak intensities (Fig. 2). Due to its better long-term stability in NMR experiments, SMRT (1255–1452) was selected for subsequent studies.

**Figure 3:**
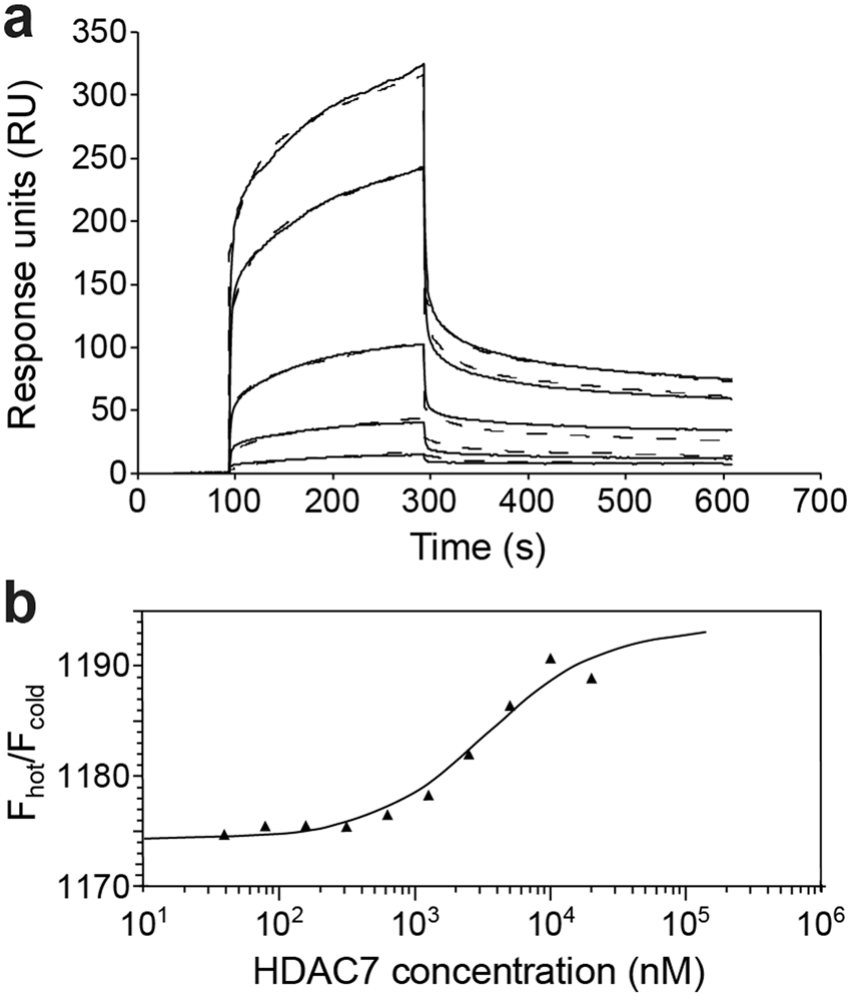
Determination of SMRT(1255–1452)–HDAC7 binding affinity. (**a**) Surface plasmon resonance curves of 8, 6, 2, 1, and 0.5 µM HDAC7 (upper to lower curve) binding to immobilized SMRT(1255–1452), yielding a K_d_ of 2.1 µM. Dashed curves represent fits to the data (solid lines) using a three-state (two-step) binding model. (**b**) Microscale thermophoresis data of HDAC7 binding to fluorescently labeled SMRT. The solid line represents a fit to the data yielding a K_d_ of 2.8 µM. Vertical axis shows the ratio of fluorescence (F) measured with and without laser heating.

The interaction of HDAC7 and SMRT (1255–1452) was measured by surface plasmon resonance (SPR) (Fig. 3a). The data could not be analyzed using a simple 1:1 binding model; however, a high quality global fit was obtained using a two-step (three-state; A + B $\leftrightharpoons$  AB(initial) $\leftrightharpoons$ AB(final)) reaction model, consistent with the interaction occurring via an initial non-specific encounter complex and/or a folding-upon-binding mechanism as described for other IDPs^46, 47^. The overall K_d_ of the interaction was measured as 2.1 µM (Table S1). Using microscale thermophoresis (MST)^48^ as a complementary solution-based method, we measured a K_d_ of 2.8 µM for SMRT(1255–1452)-HDAC7 binding (Fig. 3b). Isothermal titration calorimetry (ITC) measurements yielded a slightly higher K_d_ of 14.1 µM, which can be rationalized by the visible precipitation of HDAC7 which hampered ITC experiments at higher concentrations. Taken together, our data thus situate the K_d_ of the interaction in the 2–3 µM range.

Based on this K_d_ value, we measured HDAC7 deacetylase activity in the presence of SMRT (1255–1452) at a concentration where most HDAC7 would be bound ([HDAC7]:[SMRT(1255–1452)] = 8 nM:10 µM) in order to test whether the reported cryptic enzymatic activity on acetyllysine substrates^6^ could be enhanced. Neither SMRT(1255–1452) nor any of the other purified fragments increased the activity of HDAC7 on acetyllysine or trifluoroacetyllysine substrates (Supplementary Fig. S3). Similar negative results have been reported for other SMRT peptides^49^, consistent with the view that SMRT-mediated deacetylation *in vivo* is performed by HDAC3.

### Structural characterization of SMRT(1255–1452)

**Figure 4:**
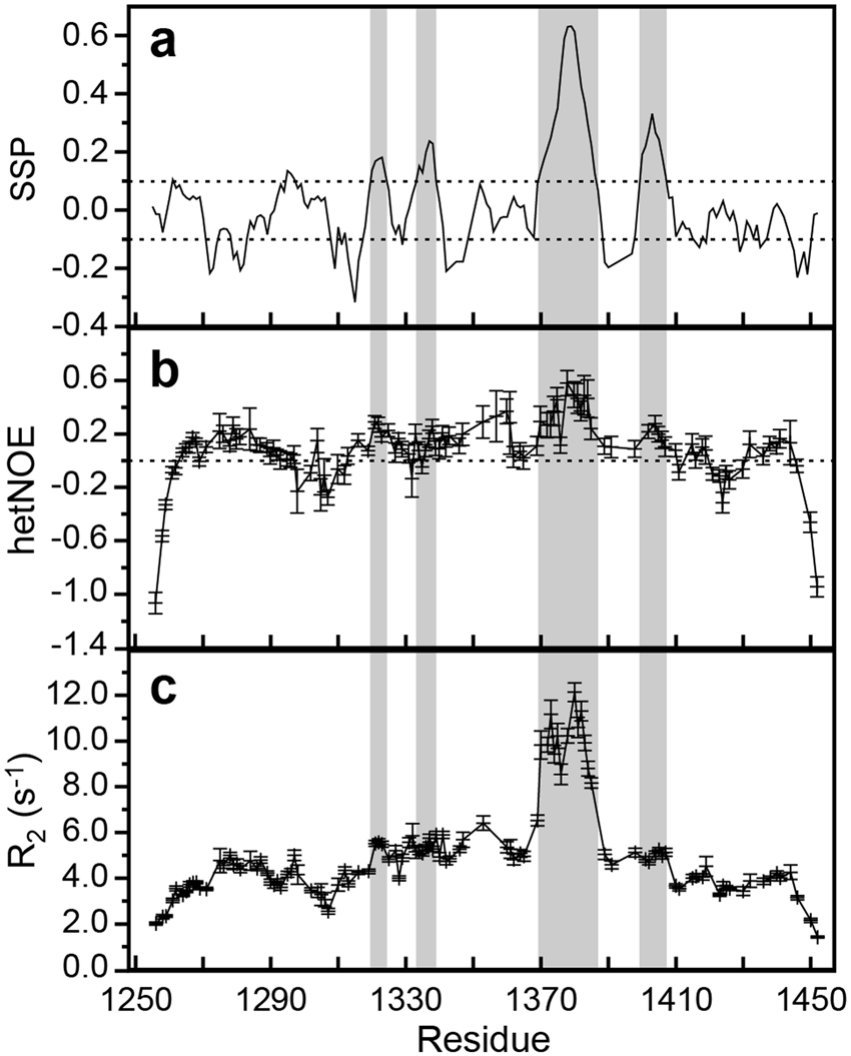
NMR analysis of SMRT(1255–1452). (**a**) Secondary structure propensity (SSP) values based on experimental Cα and Cβ chemical shifts. Positive values indicate α-helical, negative values β-extended propensity. Cutoff values of 0.1 are indicated by horizontal dashed lines. (**b**) ^1^H-^15^N heteronuclear nuclear Overhauser enhancement (hetNOE) values at 25 °C and 600 MHz ^1^H Larmor frequency. (**c**) Transverse (*R*_2_) ^15^N relaxation rates measured at 25 °C and 600 MHz ^1^H Larmor frequency. Regions with SSP values above 0.1 and elevated hetNOE values, indicative of transient helical structure, are indicated with gray shading. Error bars in panels (b) and (c) represent parameter standard deviations estimated based on noise levels in NMR spectra. Residues without data in panels (b) and (c) are prolines (which do not appear in HSQC-type spectra), have overlapping resonance signals, or are unassigned due to insufficient signal intensity.

We obtained NMR resonance assignments for 97% of the assignable sequence of SMRT(1255–1452) using standard BEST-type triple-resonance experiments^50^ on a ^13^C,^15^N-labeled sample. We used the resultant chemical shifts and transverse ^15^N relaxation rates (R_2_) and {^1^H}-^15^N heteronuclear nuclear Overhauser enhancements (hetNOEs) to identify regions of transient secondary structure (Fig. 4). Secondary structure propensity (SSP)^51^ calculated from chemical shifts exhibits elevated positive values in four continuous stretches of SMRT(1255–1452), namely residues 1320–1324, 1334–1338, 1370–1386 and 1400–1407, indicating transient helical structure (Fig. 4a). HetNOE values are also elevated in these regions, consistent with reduced mobility on the pico- to nanosecond timescale due to transient structure formation (Fig. 4b). For residues 1370–1386 whose SSP values are particularly large, a sizable increase of transverse ^15^N relaxation rates, with a periodicity of 3 to 4 residues, is also apparent (Fig. 4c), further supporting the existence of helical structure propensity in this region. Notably, all four transiently helical regions are preceded by Asp or Ser residues, which can N-cap α-helices by accepting hydrogen bonds from free amides via their sidechains^52, 53^. Several examples of transient helices in disordered proteins that are initiated by N-capping residues have been described^54–56^, suggesting that the presence of transient helical structure in SMRT is encoded in its primary sequence.

### Identification of the HDAC7 binding site of SMRT by NMR, mutagenesis and truncation

**Figure 5:**
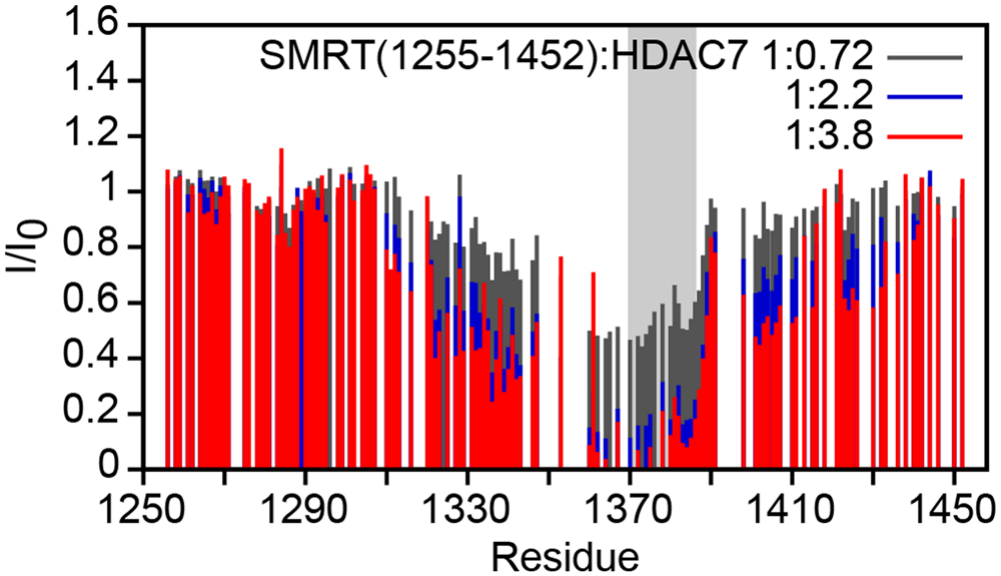
Interaction of SMRT(1255–1452) with HDAC7. Bar plots of relative signal intensities (HDAC7-complexed vs. free) for SMRT(1255–1452) residues in HSQC spectra with different amounts of added wt-HDAC7 (see Supplementary Fig. S4). The longest region of transient helical structure in free SMRT(1255–1452) (residues 1370–1386) is indicated by gray shading. Missing bars correspond to proline residues, residues whose HSQC peaks overlap, or unassigned residues.

We used SMRT(1255–1452) resonance assignments to identify the HDAC7 binding site. SMRT(1255–1452) was titrated with increasing amounts of unlabeled HDAC7, and the attenuation of peak intensities in HSQC spectra was monitored. We observed a clear and systematic decrease in intensity in a large region in the center of the molecule (residues 1310 to 1430), with the strongest attenuations observed in residues 1360–1387 (Fig. 5, Supplementary Fig. S4). These data suggest that the region of residues 1370–1386, containing the most pronounced pre-formed transient helical structure, binds HDAC7 and becomes extended to a longer helix, again consistent with a folding-upon-binding interaction^46, 57^. The actual binding most likely occurs in the strongly attenuated residues 1360–1387, contained in one of the predicted protein interaction sites of SMRT (Supplementary Fig. S1). We investigated this hypothesis by fitting the dependence of SMRT(1255–1452) peak intensities on the HDAC7 concentration to obtain an estimate of the K_d_. While this method does not allow for a precise K_d_ determination, it yields micromolar values approaching those obtained from SPR, MST and ITC for residues 1360–1386 (<40 µM; residue 1387 could not be included in this analysis due to peak overlap), but not for residues outside of this region (Supplementary Fig. S5), indicating that residues 1360–1387 constitute the minimal HDAC7 interacting region of SMRT(1255–1452).

**Figure 6:**
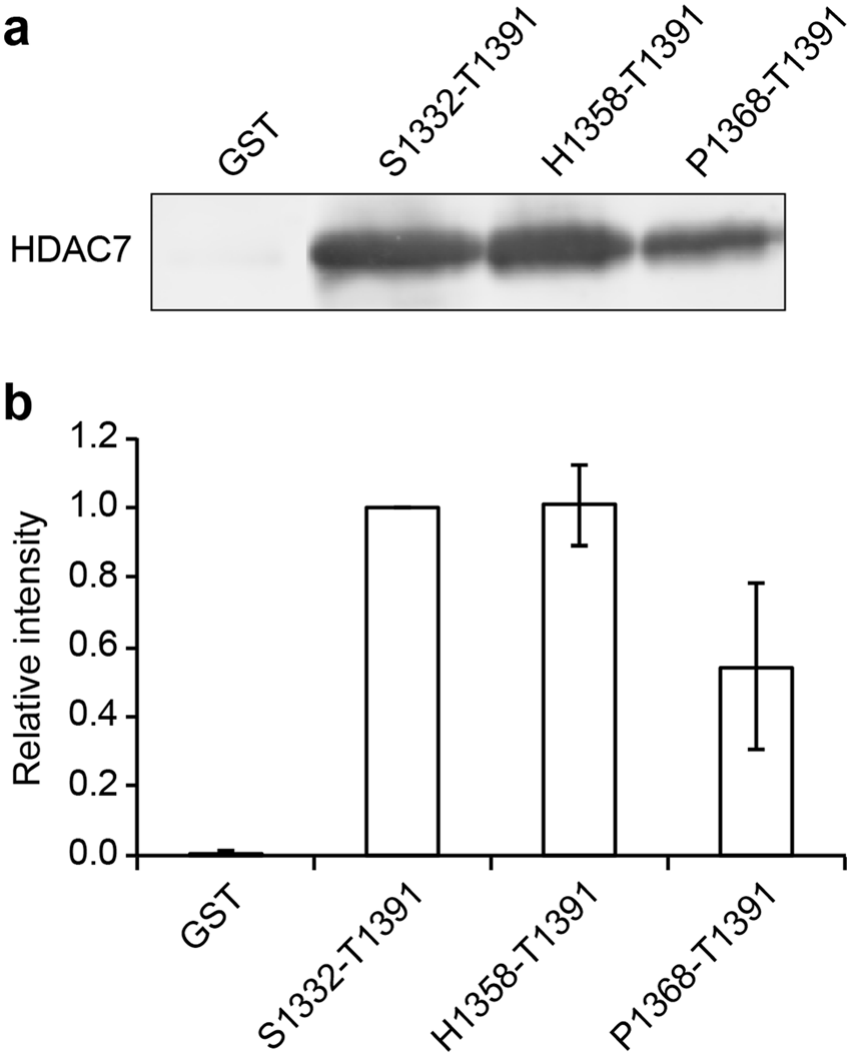
HDAC7 pull-down assays with GST-SMRT peptides derived from the SMRT(1255–1452) binding region. (**a**) Anti-hexahistidine tag western blot of HDAC7 binding to the three peptides. (**b**) Fluorescence quantification of relative intensity of HDAC7 signals shown in panel (a), with intensity for the S1332-T1391 construct set to 1. Error bars show standard deviations from 3 experiments.

Binding of this SMRT region to HDAC7 was further verified by pull-down experiments using GST-linker fusions with SMRT peptides comprising residues 1368–1391, 1358–1391, and 1332–1391. For comparison, we used a control construct comprising GST with the same linker, plus an unrelated 13-mer encoded by the translated polylinker of the parental pGEX-6p-1 vector. All three SMRT-derived peptides interact with HDAC7, whilst the control shows no binding. The longer SMRT constructs 1332–1391 and 1358–1391 bind at similar levels, while binding is reduced by about half in the shortest construct 1368–1391 (Fig. 6). This again suggests that the minimal interacting region is located within the SMRT(1358–1391) sequence, in agreement with the NMR data.

**Figure 7:**
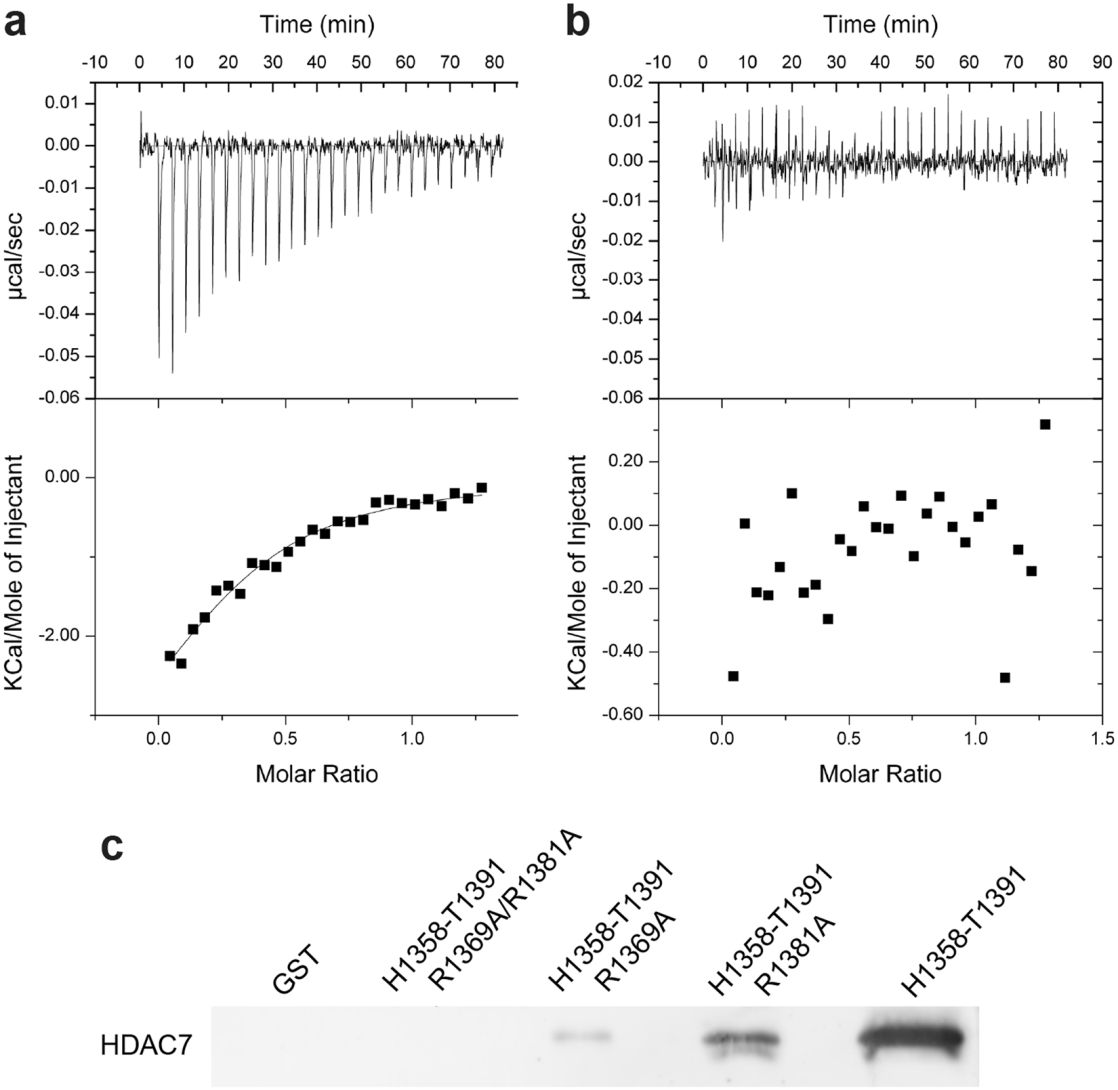
HDAC7 binding to SMRT(1255–1452) alanine mutants. (**a**,**b**) Isothermal titration calorimetry data of the HDAC7 interaction with the wild-type SMRT(1255–1452) construct (**a**) and the SMRT(1255–1452) R1369A mutant (**b**). (**c**) Western blot of pull-down assay testing HDAC7 binding of the wild-type SMRT(1358–1391) GST fusion peptide as well as its mutants R1369A, R1381A and the double mutant R1369A/R1381A.

To identify SMRT residues responsible for the interaction, we mutated bulky charged or hydrophobic residues of SMRT(1255–1452) that showed strong reductions in peak intensity in NMR spectra upon HDAC7 binding. Mutants R1360A, R1369A, V1372A, E1376A, L1379A, R1381A, K1384A and L1386A were tested for HDAC7 binding by ITC, revealing R1369A to strongly disrupt the interaction (Fig. 7a,b). Other mutants had little effect on binding, with the largest change observed for R1381A, which reduced affinity approximately four-fold (Table S2). Precise determination of K_d_ values in ITC experiments was again hampered by aggregation at higher HDAC7 concentrations; thus, the R1369A and R1381A mutants were also analyzed by pull-down assays in the context of the minimal GST-SMRT (1358–1391) peptide fusion (Fig. 7c). In agreement with ITC data, R1369A strongly reduced binding. The R1381A mutation exhibited a smaller effect (again a reduction by approximately four-fold), while the double mutation R1369A/R1381A eliminated the interaction. These results confirm that SMRT residues 1358–1391 cover the minimal HDAC7 interacting region and that R1369 and, to a lesser extent, R1381, contribute directly to HDAC7-SMRT binding.

### Mapping of the SMRT binding site of HDAC7

**Figure 8:**
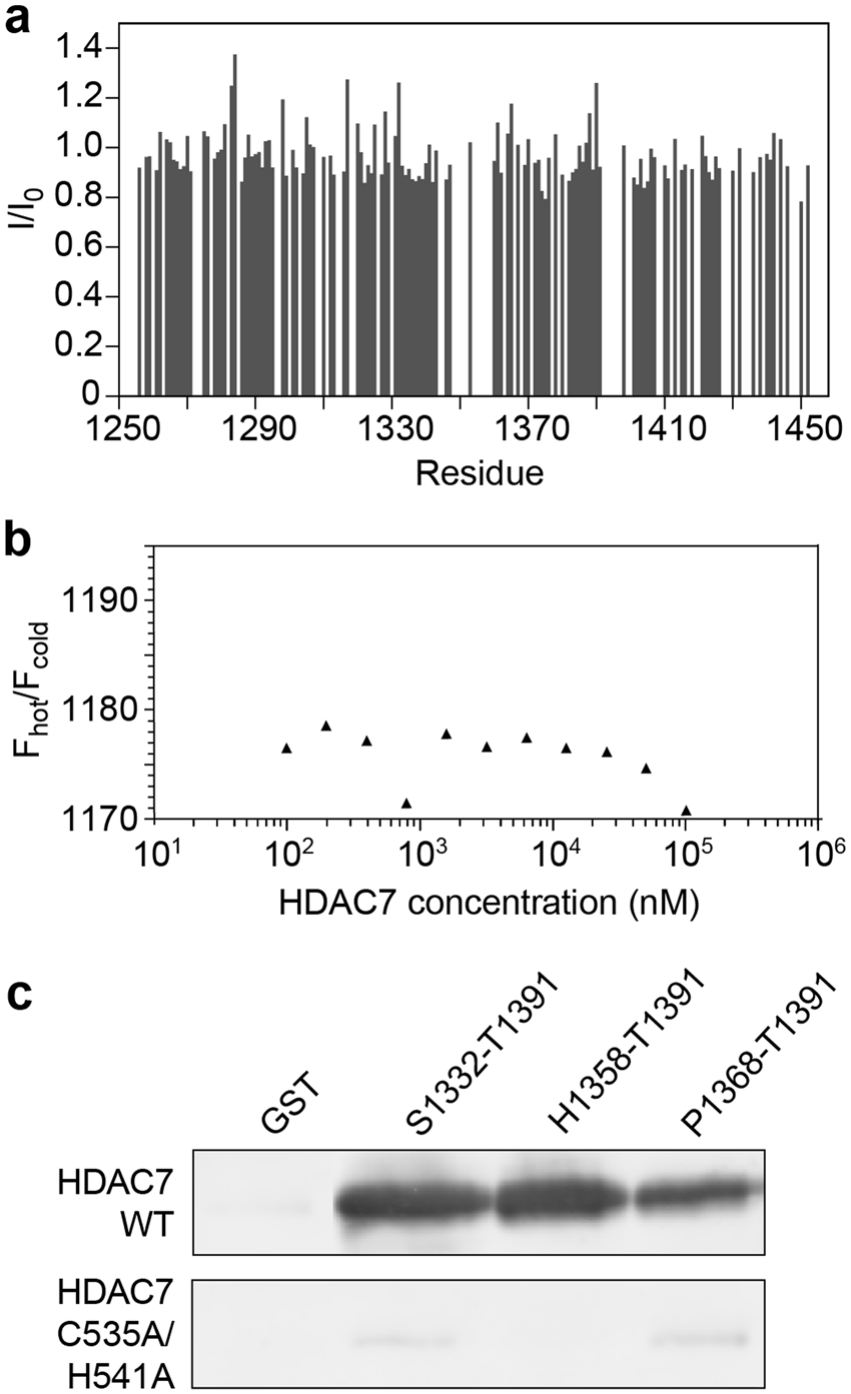
Test of SMRT(1255–1452) binding to the C535A/H541A mutant of HDAC7. (**a**) Intensities of resonance signals of SMRT(1255–1452) residues in ^15^N-^1^H HSQC NMR spectra with added 3.6-fold molar excess of the HDAC7 (C535A/H541A) mutant, normalized to corresponding intensities in the HSQC spectrum of free SMRT(1255–1452). Compare to Figure 5 for WT HDAC7. Missing bars correspond to proline residues, residues with overlapping resonance signals, or unassigned residues. (**b**) Microscale thermophoresis measurement of the interaction of SMRT(1255–1452) with the HDAC7 (C535A/H541A) mutant. See Fig. 3b for WT HDAC7. (**c**) Western blot of pull-down assay testing HDAC7 (C535A/H541A) binding to the three GST fusion peptides derived from SMRT(1255–1452). Results of the same assay for WT HDAC7 are reproduced from Fig. 6a for comparison.

To test the influence of the HDAC7 surface zinc ion on the interaction with SMRT, we mutated two of the residues involved in its coordination (C535, H541) to alanines. No intensity changes were observed in HSQC NMR spectra of SMRT(1255–1452) with HDAC7 (C535A/H541A) added in fourfold excess (Fig. 8a). In MST experiments, the thermophoretic behavior of SMRT(1255–1452) remained unchanged upon addition of HDAC7 (C535A/H541A) (Fig. 8b), and GST fusion peptides comprising subregions of SMRT(1255–1452) did not interact with HDAC7 (C535A/H541A) in pull-down assays (Fig. 8c). These data confirm a role of the HDAC7 surface zinc binding site in SMRT binding, as also observed in HDAC4^7, 49, 58^.

## Discussion

We used a random library screening technology (ESPRIT), originally devised to delineate domain boundaries in folded proteins^44^, to address the problem of identifying well-behaving constructs in a domainless hydrophilic polypeptide, where stability is not defined by the burial of hydrophobic residues, but by resistance to endogenous proteolysis. In this divide-and-conquer manner, we obtained multimilligram quantities of overlapping fragments of the large disordered SMRT co-repressor for use in interaction studies. Using NMR spectroscopy, we were able to identify a stretch of 28 residues within a 900-residue region of SMRT as a HDAC7 binding site. We confirmed the interaction by SPR, MST, and ITC, measuring a dissociation constant of 2–3 µM. We have mapped the binding interface in detail, showing that it comprises a region of pre-formed transient helical structure in SMRT, with two arginine residues (R1369, R1381) crucial for the interaction, as well as the HDAC7 surface zinc binding site.

Pre-existing secondary structure has been described in a variety of IDPs and has in many cases been implicated in binding of IDPs to their biological partners^46, 47, 56, 59^. Our results for the SMRT-HDAC7 interaction strengthen this notion by showing that part of the HDAC7 binding site of SMRT is helically preconfigured. The entire core binding site (residues 1360–1387) extends N-terminally beyond the region of pre-formed helical secondary structure, as also observed in the binding interaction of the phosphoprotein and nucleoprotein in vesicular stomatitis virus^57^. Together with SPR data indicating a two-step binding mechanism, this finding suggests that SMRT binds HDAC7 via an induced-fit mechanism where the final bound conformation of SMRT is only obtained after structural rearrangements in an initial encounter complex. However, structural information on the bound SMRT conformation as well as kinetic data would be required to confirm this hypothesis^60^. Nevertheless, our data are suggestive of a two-step, folding and binding mechanism for the HDAC7-SMRT interaction. The intensity reduction observed in NMR signals from residues flanking the binding site may be due to a reduction in mobility of chain segments adjacent to the binding site, or caused by transient interactions occurring in formation of the initial encounter complex, as described in the “fly-casting” hypothesis^61^. The measured K_d_ of 2–3 µM is typical for binding interactions of peptide motifs in disordered proteins and fits well with the purpose of the complex in signalling, where an overly strong association might interfere with its necessarily transient nature^27, 62, 63^.

A recent study revealed an imperfectly repeated 8 amino acid sequence dubbed the “GSI motif”, occurring five times within the RD3 of SMRT, as being crucial for binding to class IIa HDACs^49^ (Supplementary Fig. S6). A 20-residue peptide containing the consensus motif comprising SMRT residues 1457–1464 was observed to bind all four class IIa enzymes with affinities of 4–8 µM. Consistent with these results, the HDAC7 binding site we identified in SMRT (residues 1360–1387) comprises one of the GSI motifs in its N-terminal region (residues 1361–1368). However, we observed directly by NMR that the pre-formed transient helical secondary structure in SMRT residues 1370–1386 also plays a role in HDAC7 binding to this site in SMRT. During pull-down experiments, a SMRT peptide (1368–1391) comprising the helical element, but without a GSI motif, bound HDAC7, albeit less tightly. Conversely, mutation of arginine residues outside of the GSI motif (R1369, R1381) reduced or abolished binding of SMRT(1255–1452), even though the GSI motif was still present. Notably, in ref. 49, among the five GSI motifs actually occurring in SMRT, only the consensus sequence (residues 1457–1464) was directly tested for binding to class IIa HDACs; the mutants investigated differ in sequence from the other four natural SMRT GSI motifs. However, it was found that even a conservative mutation of T7 in the consensus motif (T7S) abolishes binding; thus, it remains to be demonstrated whether three of the five SMRT GSI motifs, which contain T7I or T7A mutations with respect to the consensus, could actually bind to class IIa HDACs on their own. Our observation that mutation of R1369 outside of the GSI motif in residues 1361–1368 abolishes binding suggests that this GSI motif, containing the T7I mutation, alone is indeed insufficient for binding, although it clearly contributes to the interaction in the presence of the following arginine and the transient helical element.

Taken together, there is direct experimental evidence for two micromolar-affinity binding sites for class IIa HDACs in SMRT, in residues 1360–1387 and 1457–1464, both containing GSI motifs. We suggest that the GSI motif in the former binds weakly due to a T7I substitution, but that compensating additional affinity is provided by the downstream helix. The latter GSI site, conforming to the consensus sequence, is sufficient for binding alone. Up to three other GSI motifs might additionally contribute to binding. Among these five sites, the one described here appears to be unique in containing a pre-formed transient α-helical element, since no other GSI motif in RD3 is predicted to be followed by helical secondary structure (Supplementary Fig. S7). Interestingly, the corresponding alignment-related GSI motif in N-CoR1 (residues 1294–1301) may also be followed by helical secondary structure, according to bioinformatic prediction (Supplementary Fig. S8). We hypothesize that the presence of two or more class IIa HDAC binding sites in RD3 of SMRT may play a role in increasing the encounter rate (k_on_) of the interaction, possibly through formation of a “fuzzy complex” where multiple sites of a disordered protein bind to a single site on the partner protein in rapid exchange^64^.

In summary, we have identified and described at single residue resolution a class IIa HDAC binding site in SMRT repression domain 3, comprising a transient α-helix preceded by a GSI motif, that HDAC7 interacts with via its surface zinc binding site. Key to the obtention of this result was the use of ESPRIT combinatorial screening in order to obtain stable, soluble constructs of the large disordered SMRT protein, coupled with a solution-based structural and biophysical approach. In common with other studies on class IIa HDACs, our results support a model whereby catalytically inactive, or cryptically active, HDAC7 is recruited into the SMRT repressor complex whose deacetylase activity is conferred by adjacently bound HDAC3^11^. Our data provide a framework for inhibition of the SMRT-HDAC7 interaction, which has therapeutic potential for disease conditions in which HDAC7 has been implicated^36, 65^ and provides a means to target type IIa HDAC function beyond the relatively conserved active sites of this enzyme class.

## Methods

### Identification of SMRT fragments using ESPRIT

The DNA encoding residues 1089–1993 of human SMRT (UniProt ID: Q9Y618) was cloned into a vector encoding an N-terminal hexahistidine tag and a C-terminal biotin acceptor peptide. This vector contains, at each end of the insert, pairs of restriction sites that leave exonuclease III-sensitive 5′ overhangs towards the insert (*Asc*I and *Not*I) and resistant 3′ overhangs towards the respective tag (*Aat*II and *Nsi*I). After restriction digest with *Aat*II and *Asc*I at the 5′ side of the insert, the exonuclease III reaction was initiated wherein 60 × 2 µl aliquots were taken at 1 min intervals and pooled in a quenching solution of 2 M NaCl on ice. After DNA clean up using a spin column (Machery Nagel), single strand overhangs were removed with mung bean nuclease, a further clean up step performed, and the vectors blunt-ended with *Pfu* polymerase prior to recircularization with T4 DNA ligase. The ligation was recovered into *E*. *coli* Mach1 cells (Thermo Scientific) and plasmid purified from about 30,000 pooled colonies. The process was repeated on this plasmid mix using *Nsi*I and *Not*I to obtain a bidirectionally truncated construct library following recovery of the library (~50,000 colonies) and plasmid preparation. *E*. *coli* BL21-AI (Thermo Fisher) was transformed and plated on agar for robotic colony picking. Approximately 28,000 clones were isolated of which one-ninth were predicted to be in frame with the N-terminal hexahistidine tag and the C-terminal biotin acceptor peptide. All clones were arrayed robotically onto nitrocellulose membranes over agar and allowed to grow overnight at room temperature. The membranes were then transferred to fresh agar at 30 °C containing 13 mM arabinose and 50 μM biotin to induce protein expression, with colonies being grown for a further 4 h, then lyzed using a SDS lysis protocol^66^. A test was performed for soluble intact protein expression using a high throughput fluorescence assay whereby clones exhibiting detectable N-terminal hexahistidine signals were ranked for biotinylation levels using Anti-His antibody (GE Healthcare) with goat anti-mouse Alexa Fluor 532 conjugate (Thermo Fisher Scientific) and streptavidin Alexa Fluor 488, respectively. Ninety-six initial hits were selected from the library and expressed in small-scale cultures, then purified on Ni^2+^ NTA agarose beads (Qiagen). Fifty-one constructs yielded soluble purifiable protein easily detectable by SDS-PAGE with Coomassie blue staining. These were DNA sequenced to identify construct boundaries, revealing most fragments as 100 to 200 amino acids in length, consistent with the DNA insert sizes generated during library synthesis. Soluble constructs clustered in 5 main regions (SMRT residues 1089–1330, 1179–1505, 1378–1618, 1677–1804 and 1740–1993). Since yields and proteolytic stability varied between clones, six constructs (comprising residues 1122–1254, 1255–1452, 1276–1405, 1784–1993, 1798–1966 and 1882–1993, respectively) were selected for further investigation, yielding 10–20 mg of purified protein per liter during scale-up experiments.

### Expression, purification and isotopic labeling of SMRT fragments

*E*. *coli* BL21-AI cells transformed with the corresponding plasmid were grown at 37 °C until OD 0.6 and expression was induced by addition of 13 mM L-arabinose. For expression of ^15^N- or ^15^N,^13^C-labeled SMRT fragments, cells were grown at 37 °C in 4 l minimal medium supplemented with ^15^NH_4_Cl or ^15^NH_4_Cl and ^13^C_6_-glucose, respectively. Cultures were incubated overnight at 25 °C and pelleted at 6,000 rpm for 30 min. The bacterial pellet was resuspended in lysis buffer (50 mM Tris pH 8.0, 300 mM KC1, 5 mM β-mercaptoethanol, 100 μM MgCl_2_) supplemented with EDTA-free complete protease inhibitor cocktail (Roche Diagnostics, Indianapolis) and benzonase, then lyzed with a microfluidizer (M11O-L; Microfluidics Corporation, Newton, MA, USA). Cell debris was pelleted by centrifugation at 20,000 rpm for 40 min at 4 °C. The heat resistant nature of the SMRT fragments permitted a pre-purification step in which the supernatant was collected and heated in a water bath to 70–80 °C until a milky white precipitate formed in the tube. After centrifugation at 20,000 rpm for 30 min at 4 °C, the supernatant collected from this step was incubated for 2 h with 1 ml equilibrated Ni^2+^ NTA agarose beads at 4 °C. The slurry was then transferred to a gravity column and washed with 150 ml wash buffer (50 mM Tris pH 8.0, 300 mM KC1, 5 mM β-mercaptoethanol) followed by elution in the same buffer supplemented with 250 mM imidazole. Eluted SMRT proteins were incubated overnight at 4 °C with TEV protease to cleave the hexahistidine tag; the TEV and cleaved tag were then removed by reverse affinity chromatography. The product was analyzed by SDS-PAGE to confirm tag cleavage and purity. The purified proteins were buffer exchanged into phosphate-buffered saline (PBS) pH 6.5 (50 mM KH_2_PO_4_/K_2_HPO_4_ pH 6.5, 150 mM KCl, 1 mM dithiothreitol (DTT)) by overnight dialysis using a Slide-A-Lyzer cassette (ThermoScientific), then concentrated to 1 mM with an Amicon Ultra centrifugal filter unit (Millipore). Proteins were either used directly or frozen in aliquots using liquid nitrogen for storage at −80 °C.

For pull-down studies, cells expressing GST fusion proteins were grown at 37 °C in LB medium to OD 0.6, then induced with L-arabinose overnight at 25 °C. Cells were harvested by centrifugation at 6,000 rpm for 30 min, resuspended in lysis buffer (25 mM HEPES pH 7.5, 200 mM NaCl, 10% glycerol, 3 mM DTT, 0.5% Tween 20, EDTA-free protease inhibitor cocktail (Roche Diagnostics), benzonase) and lyzed by sonication. The lysate was centrifuged at 20,000 rpm for 30 min at 4 °C, the supernatant was mixed with 4 ml equilibrated glutathione sepharose beads (GE Healthcare) and incubated at 4 °C for 2 h to allow binding. Beads were sedimented by centrifugation at 500 rpm for 5 min and washed with 5 resin volumes of binding buffer (PBS pH 7.3, 3 mM DTT). For elution, 2 ml of elution buffer (50 mM Tris-HCl pH 8.0, 10 mM reduced glutathione) were added to each sample and incubated 30 min at 4 °C before sedimenting the beads and removing the supernatant containing purified GST-fusion proteins. Proteins were dialyzed overnight into binding buffer using Slide-A-Lyzer cassettes (ThermoScientific) and concentrated to 300 µM using Amicon Ultra centrifugal filter units (Millipore) for storage. Alanine mutant GST peptides were purified with the same protocol.

### Expression and purification of recombinant HDAC7

A synthetic gene containing residues 449–952 of human HDAC7 (UniProt ID: Q8WUI4), covering the catalytic domain, was used for expression in a pET9-derived plasmid with N-terminal hexahistidine tag. Transformed *E*. *coli* BL21-AI cells were grown at 37 °C to OD 0.4, then ZnCl_2_ was added to 50 µM final concentration and, at OD 0.6, arabinose was added to 0.2% w/v and the cultures incubated overnight at 25 °C. From 12 l of culture, 80–120 g cells were harvested. Pellets were resuspended in 80 ml lysis buffer (25 mM HEPES pH 7.5, 200 mM KCl, 10% glycerol, 0.5% Igepal, 1 mM DTT) supplemented with benzonase, EDTA-free protease inhibitor cocktail (Roche Diagnostics) and lyzed by sonication. Glycerol was then added to the lysate to a final concentration of 30% v/v and cell debris removed by centrifugation at 20,000 rpm, 40 min, 4 °C. The supernatant was collected and incubated overnight at 4 °C with 8 ml Zn^2+^ NTA beads (Qiagen). Beads were then washed twice with 1 l of wash buffer (buffer 1: 25 mM HEPES pH 7.5, 1 M KCl, 10% glycerol, 0.1% n-octyl-β-D-glucopyranoside, 1 mM DTT, 5 mM imidazole; buffer 2: 25 mM HEPES pH 7.5, 200 mM KCl, 10% glycerol, 0.1% n-octyl-β-D-glucopyranoside, 1 mM DTT, 20 mM imidazole) in a gravity column. Elution was performed with an imidazole concentration gradient proceeding stepwise from 50 to 500 mM. Fractions were analyzed by SDS-PAGE and the samples containing protein were pooled. The hexahistidine tag was removed by overnight incubation at 4 °C with TEV protease. The sample was concentrated using an Amicon Ultra centrifugal filter unit (Millipore), then purified further by anion exchange chromatography using a Q sepharose column (GE Healthcare). Elution was achieved with a step gradient of high salt buffer containing 25 mM HEPES pH 7.5, 10% glycerol, 1 mM DTT, 0.1% n-octyl-β-D-glucopyranoside, 1 M KCl. Fractions containing HDAC7 were pooled and concentrated using an Amicon Ultra centrifugal filter unit (Millipore) to a volume of 30 ml prior to overnight dialysis in PBS pH 6.5. After dialysis, the sample was again concentrated to 20–50 µM and used directly or stored at −80 °C.

### Surface plasmon resonance (SPR)

The interaction between SMRT(1255–1452) and wt-HDAC7 was measured on CM5 sensor chips using a Biacore 3000 system (GE Healthcare). The surface of the chips was first activated with 70 µl of 200 mM EDC and 50 mM NHS in a 1:1 ratio at a flow rate of 10 µl/min. Then, SMRT(1255–1452) was injected at a concentration of 30 µg/ml in 10 mM sodium acetate pH 5.5 until 5500 RU of protein was immobilized. Remaining active esters were deactivated by injecting 70 µl of 1 M ethanolamine/hydrochloride, pH 8.5. After ligand immobilization, the chip was equilibrated in running PBS buffer (50 mM KH_2_PO_4_/K_2_HPO_4_ pH 6.5, 100 mM KCl, 1 mM DTT). To measure interactions, HDAC7 was injected at concentrations from 500 nM to 8 µM at a flow rate of 20 µl/min for 300 s. Dissociation was measured over 400 s, after which two consecutive washes with 30 µl 2 M MgCl_2_ were performed to regenerate the chip.

Data were analyzed using the software BIAevaluation 3.0. A simple 1:1 Langmuir binding model did not fit the data, but we reasoned that a two-step (three-state) reaction model (Equation 1) should be used for data fitting as described elsewhere for IDP interactions^46, 47, 67–69^. This assumes binding occurs via an intermediate that undergoes a conformational change to generate a stable complex. Two association and two dissociation constants are calculated with this model, from which the overall apparent K_d_ can be derived (Equation 2).

1 $${\rm{A}}+{\rm{L}}\underset{{k}_{d1}}{\overset{{k}_{a1}}{\rightleftharpoons }}{\rm{AL}}\underset{{k}_{d2}}{\overset{{k}_{a2}}{\rightleftharpoons }}{{\rm{AL}}}^{\ast }$$

2 $${K}_{d}=\,\frac{{k}_{d1}{k}_{d2}}{{k}_{a1}({k}_{a2}+{k}_{d2})}$$

### Microscale Thermophoresis (MST)

The interactions of SMRT(1255–1452) with the HDAC7 catalytic domain and HDAC7 C535A/H541A was studied using a Monolith NT.115 instrument (NanoTemper technologies). A 20 µM solution of SMRT(1255–1452) in PBS buffer was fluorescently labeled using an NT.115 labeling kit RED NHS Amine Reactive Dye NT647 (NanoTemper Technologies), followed by removal of excess dye and buffer exchange using PD10 columns (Amersham). A 1:2 serial dilution of both wt-HDAC7 and HDAC7 C535A/H541A in 25 mM Tris pH 8.0, 10 mM MgCl_2_, 150 mM KCl and 0.5% Tween-20 was prepared, resulting in concentrations ranging from 20 nM to 20 µM. Labeled SMRT(1255–1452) was added to each dilution and briefly incubated before loading the samples onto hydrophilic capillaries. Measurements were taken at room temperature. Results were analyzed using MO Affinity analysis software (NanoTemper Technologies).

### Isothermal titration calorimetry (ITC)

SMRT(1255–1452) wild-type and alanine mutants were tested for binding to wild-type HDAC7 using an ITC200 microcalorimeter. The wild-type HDAC7 and the SMRT(1255–1452) mutants were dialyzed into PBS (50 mM KH_2_PO_4_/K_2_HPO_4_ pH 6.5, 100 mM KCl, 1 mM DTT) with 1 mM β-ME. HDAC7 was placed in the cell at 25 °C at a concentration of 50 µM for each titration. Twenty-six 1.5 µl injections of the SMRT(1255–1452) alanine mutants at a concentration of 300 µM were then performed at 180 s intervals. Data were analyzed using Origin 7.0 software (MicroCal USA). The baseline of each measurement was first adjusted manually before integration.

### Pull-down assays

GST fusions with peptides P1368-T1391, H1358-T1391, and S1332-T1391 from the HDAC7 binding region of SMRT(1255–1452) were analyzed for binding to HDAC7 using pull-down assays. Fifty microliters of glutathione sepharose 4 fast flow bead slurry (GE Healthcare) were used per pull-down assay. The beads were equilibrated in binding buffer (PBS pH 7.3, 3 mM DTT), incubated for 2 h with 0.3 mg of purified GST-peptide or GST control, and washed in 5× bead volume of binding buffer to remove unbound protein. The beads were blocked by incubation in 300 µl SuperBlock PBS Blocking Buffer (Thermo Scientific) for 1 h. This was followed by a 2 h incubation with supernatant from an *E*. *coli* BL21 AI RIL HDAC7 expression lysate at 4 °C. The beads were washed 6 times with 1 ml wash buffer (1x PBS pH 7.3, 3 mM DTT, 0.05% Tween 20, 500 mM NaCl), each time sedimenting the beads by centrifugation at 600 rpm for 3 min. Elution of proteins from beads was performed by addition of 25 µl GST elution buffer (50 mM Tris-HCl pH 8.0, 10 mM reduced glutathione), followed by 1 h of incubation. Samples were analyzed by SDS-PAGE and western blotting to detect the presence of HDAC7 using an anti-His antibody (GE Healthcare) and goat anti-mouse Alexa Fluor 532 conjugates (Thermo Fisher Scientific). Bands on western blots were visualized with a Typhoon Trio imager (GE Healthcare) and quantified using ImageQuant software (GE Healthcare). The same protocol was applied for HDAC7 C535A/H541A and mutants R1369A, R1381A and R1369A/R1381A of the peptide H1358-T1391.

### Test of HDAC7 deacetylase activity

The influence of SMRT fragments on HDAC7 deacetylase activity on two substrates, trifluoroacetyllysine and acetyllysine (BPS Biosciences) was tested over 1.5 h of incubation at 37 °C (single end point measurement). One nanogram (8 nM) of HDAC7 was incubated with 20 µM of substrate in HDAC assay buffer provided with the assay kit. SMRT fragments were added to a final concentration of 10 µM. The reaction was stopped by addition of developer solution provided with the kit and incubation for 25 min at room temperature (about 22 °C). Fluorescence intensity was measured using a Perkin Elmer Victor2 spectrophotometer at excitation and emission wavelengths of 355 nm and 460 nm, respectively.

### Nuclear magnetic resonance spectroscopy

NMR experiments were performed on Varian spectrometers operating at ^1^H Larmor frequencies of 600 and 800 MHz. Sample buffer contained 50 mM potassium phosphate, 100 mM KCl, 1 mM DTT and 10% D_2_O at pH 6.5. Spectra were recorded at 25 °C. Concentrations of SMRT(1255–1452) in assignment and relaxation experiments were between 270 and 330 μM. For titrations with wild-type HDAC7, the following SMRT(1255–1452):HDAC7 concentrations were used: 100 μM: 72 μM (1:0.72); 59 μM: 128 μM (1:2.2); 40 μM: 151 μM (1:3.8). A single mixture of SMRT(1255–1452) with the HDAC7 C535A/H541A mutant was prepared, using concentrations of 40 μM for SMRT(1255–1452) and 144 μM for the HDAC7 mutant (1:3.6). In two-dimensional ^1^H-^15^N HSQC spectra recorded at 600 MHz, typically 1024 and 125 complex points were recorded in the direct and indirect dimension, respectively, using spectral widths of 7022 and 1500 Hz. For assignment, a series of BEST-type triple-resonance experiments^50^ was employed (HNCO, intraresidue HN(CA)CO, HN(CO)CA, intraresidue HNCA, HN(COCA)CB, intraresidue HN(CA)CB). Transverse ^15^N relaxation rates (R_2_) and {^1^H}-^15^N heteronuclear nuclear Overhauser enhancements (hetNOEs) were recorded using standard pulse sequences at a ^1^H Larmor frequency of 600 MHz, using relaxation periods between 10 and 250 ms for the R_2_ measurement and a 4 s saturation or delay period for saturation and reference spectra, respectively, in the hetNOE experiment (in addition to a 2 s inter-scan delay). R_2_ and hetNOE error values were estimated based on the standard deviation of the noise in NMR spectra. Spectra were processed with NMRPipe^70^ and analyzed using Sparky^71^ and CcpNmr^72^. Automated resonance assignment of SMRT(1255–1452) was performed using the software MARS^73^, followed by manual verification.

## Electronic supplementary material

Supplementary info
