# Supplementary material for: Structural Characterization of the SMRT Corepressor Interacting with Histone Deacetylase 7
Source: Sci Rep. 2017 Jun 16;7:3678. doi: 10.1038/s41598-017-03718-5 (PMC5473869; doi:10.1038/s41598-017-03718-5)
Supplement: Supplementary file 1 — Supplementary info [file 41598_2017_3718_MOESM1_ESM.docx]

# Supplementary Information

# Structural Characterization of the SMRT Corepressor Interacting with Histone Deacetylase 7

Danielle C. Desravines, Itziar Serna Martin, Robert Schneider, Philippe J. Mas, Nataliia Aleksandrova, Malene Ringkjøbing Jensen, Martin Blackledge & Darren J. Hart


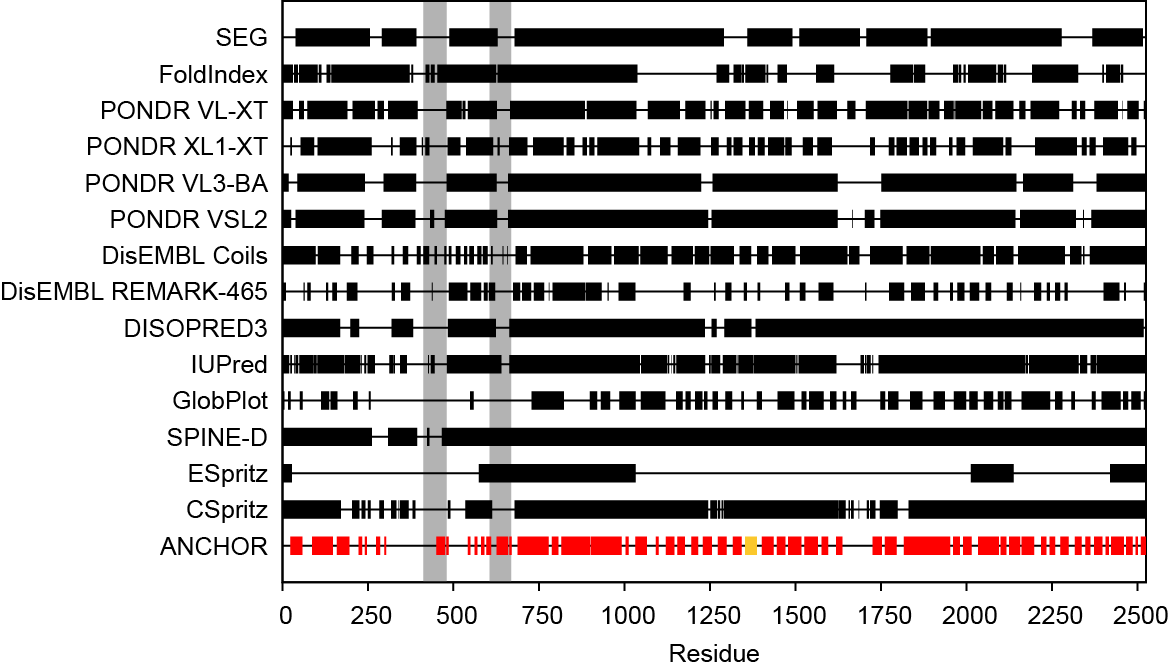


**Supplementary Figure S1:** **Predictions of disorder and interaction sites for full-length SMRT** **(UniProt ID: Q9Y618)**. The algorithms employed for disorder prediction were: SEG^1^ (http://mendel.imp.ac.at/METHODS/seg.server.html); FoldIndex^2^ (http://bip.weizmann.ac.il/
fldbin/findex); PONDR^3–5^ (http://www.pondr.com/); DisEMBL^6^ (http://dis.embl.de/); DISOPRED3^7^ (http://bioinf.cs.ucl.ac.uk/psipred/?disopred=1); IUPred^8,9^ (http://iupred.enzim.hu/); GlobPlot^10^ (http://globplot.embl.de/); SPINE-D^11^ (http://sparks-lab.org/SPINE-D/); ESpritz^12^ (http://protein.bio.unipd.it/espritz/); and CSpritz^13^ (http://protein.bio.unipd.it/cspritz/). ANCHOR^14,15^ (http://anchor.enzim.hu/) was used to predict potential protein binding sites. Default parameters as set on the corresponding websites were used, except for ESpritz (Disprot prediction, threshold 5% false positive rate) and CSpritz (Disprot prediction). The SEG result shown was obtained using a window length of 45 residues. For DISOPRED3, the SMRT sequence was split in two (residues 1-1320 and 1321-2525) to conform to the length limitations of the algorithm. Predicted regions of disorder are shown as black rectangles. Interaction sites predicted by ANCHOR are shown as red rectangles; one of these, encompassing residues 1353-1387, contains the HDAC7 binding site identified in this work (residues 1360-1387) and is shown in yellow. Gray vertical bars mark the two known structured SANT-like domains of SMRT (residues 412-480 and 606-669)^16–18^.


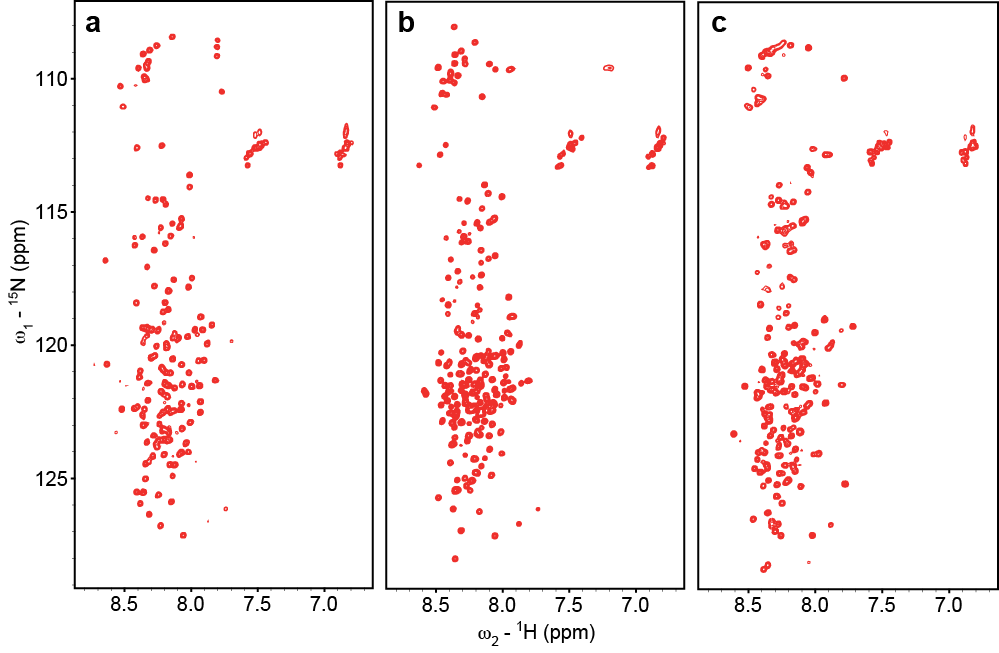


**Supplementary Figure S2: ^1^H-^15^N HSQC spectra of non-overlapping soluble constructs of SMRT.** (**a**) SMRT(1122-1254), (**b**) SMRT(1255-1452), (**c**) SMRT(1784-1993). Constructs were obtained directly from ESPRIT screening and contain C-terminal biotinylated peptide tags.

**a**


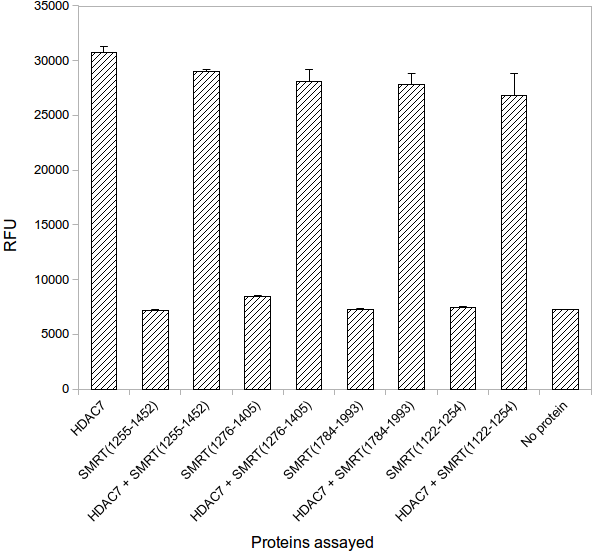


**b**


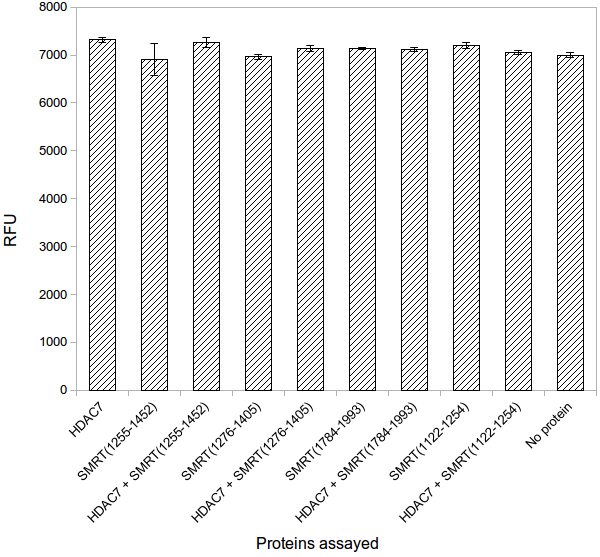


**Supplementary Figure S3: SMRT fragments do not stimulate HDAC7 deacetylase activity. (a**) Deacetylase activity of purified HDAC7 is detectable on trifluoroacetyllysine substrates. Individual SMRT fragments show no activity and do not change the level of activity of HDAC7. Of the different SMRT fragments tested, the overlapping SMRT(1255-1452) and (1276-1405) both bind HDAC7 as shown by NMR. (**b**) Activity of HDAC7 alone and with SMRT fragments on acetyllysine substrate. Activity of purified enzyme relative to background signal (no protein) and SMRT fragments alone is negligible. Addition of 10 µM SMRT fragments to HDAC7 (5 x K_D_) has no effect. RFU: relative fluorescence units.


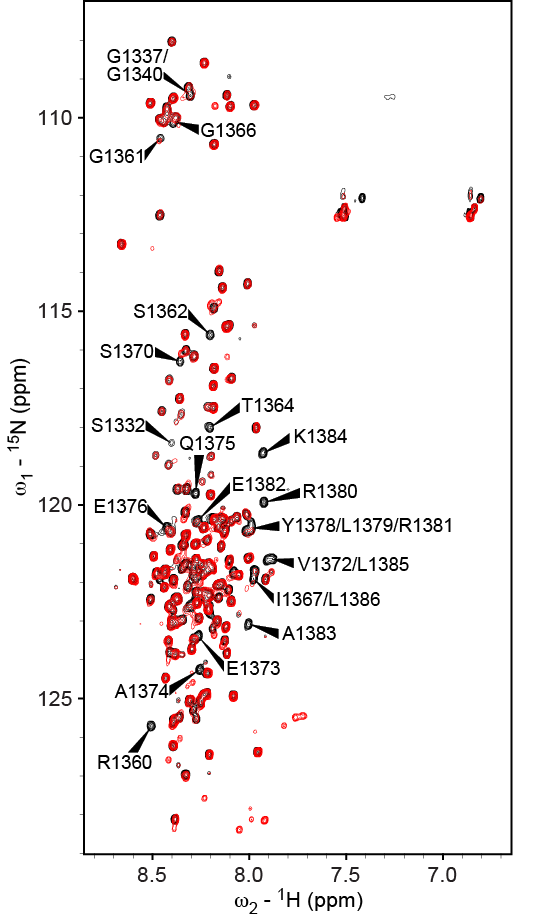


**Supplementary Figure S4: Interaction of SMRT(1255-1452) with HDAC7.** Superposition of ^15^N-^1^H HSQC spectra of free ^15^N-labeled SMRT(1255-1452) (black) and SMRT(1255-1452) with a 3.8-fold excess of unlabeled wt-HDAC7 (red). Assignments for several strongly attenuated signals are indicated. Compare plot of relative intensities in Figure 5 in the main text.


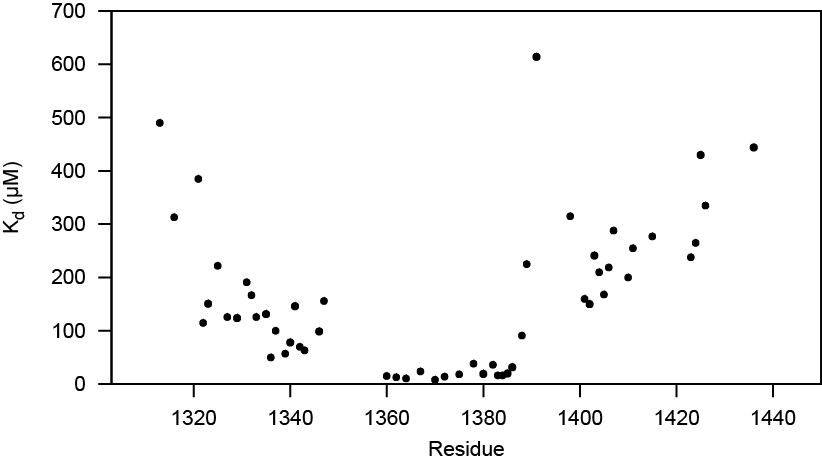


**Supplementary Figure S5: Residue-specific K_d_ estimates for binding of SMRT(1255-1452) to HDAC7.** Normalized relative peak intensities (HDAC7-complexed vs. free) in the three SMRT(1255-1452) ^15^N-^1^H HSQC spectra with 0.72, 2.2, and 3.8 molar equivalents of wt-HDAC7 (see Figure 5 in the main text) were fitted to the standard equation for determining the K_d_ of a bimolecular binding reaction from fast-exchange NMR chemical shift changes upon binding^19^. Here, relative peak intensities *I* were used instead of chemical shifts:


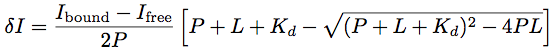


with *I*_bound_ (relative peak intensity in the fully bound state) and *I*_free_ assumed to be 0 and 1, respectively. Given the small number of titration points and the heuristic method used here in the absence of fast-exchange chemical shift changes, the results should only be taken as rough estimates. Nevertheless, the data consistently show low residue-specific K_d_ values between 8 and 38 µM for residues 1360 – 1386 and (much) higher values for all other residues, in agreement with the notion of residues 1360 – 1386 being the primary HDAC7 interaction site. A joint fit of the data from these residues yields a K_d_ of 19 µM. It should be noted that the stability of HDAC7 under the conditions of the NMR experiments was limited and formation of a precipitate was observed, as was also the case for ITC experiments, leading to lower effective concentrations of HDAC7 and, consequently, an overestimate of the K_d_ when using the nominal concentrations in the fit. With all limitations of this K_d_ estimate taken into account, the results can be considered to be in good agreement with the K_d_ values of 2 – 3 µM we obtained for the SMRT(1255-1452)-HDAC7 interaction using other biophysical methods.


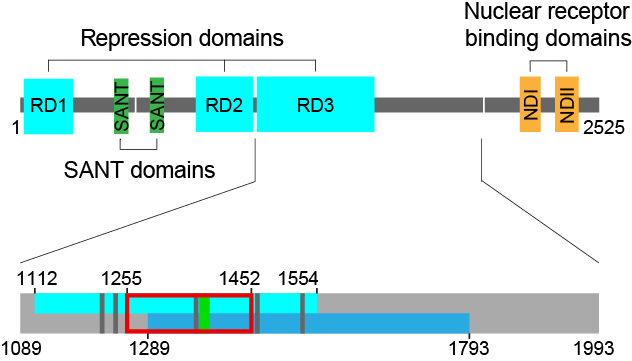


**Supplementary Figure S6: Functional and structural domains of the SMRT protein.** Compare Figure 1 in the main text. Bottom panel shows a close-up view of the region investigated in this work. Light blue, repression domain 3 (RD3); blue, HDAC7 interaction region as determined in ref. ^20^; red frame, SMRT(1255-1452) construct; dark gray vertical bars, GSI motifs as defined in ref. ^21^; light green, transient pre-formed helical region as found by NMR (residues 1370-1386).

1213 1223 1233 1243

RGTALGSVPG GSITKGIPST RVPSDSAITY RGSITHGTPA

Agadir ---------- ---------- ---------- ----------

Jpred ---------- --EE------ ---------- ----------

Psipred ---------- ---------- ---------- ----------

1234 1244 1254 1264

VPSDSAITYR GSITHGTPAD VLYKGTITRI IGEDSPSRLD

Agadir ---------- ---------- ---------- ----------

Jpred ---------- ---------- ---------- ----------

Psipred ---------- ---------- ------EEEE ----------

1360 1370 1380 1390

HHLKEQHHIR GSITQGIPRS YVEAQEDYLR REAKLLKREG

Agadir ---------- ---------- HHHHHHHHHH HHHHHH----

Jpred ---------- ---------- ---------- ----------

Psipred ---------- ---------- -HHHHHHHHH HHHHHHH---

1456 1466 1476 1486

LPLAPRPLKE GSITQGTPLK YDTGASTTGS KKHDVRSLIG

Agadir ---------- ---------- ---------- ----------

Jpred ---------- ---------- ---------- ----------

Psipred ---------- ---------- ---------- ----------

1527 1537 1547 1557

SRPGTASSSG GSIARGAPVI VPELGKPRQS PLTYEDHGAP

Agadir ---------- ---------- ---------- ----------

Jpred ---------- ---------- ---------- ----------

Psipred ---------- ---------- ---------- ----------

**Supplementary Figure S7: Secondary structure predictions for the regions of SMRT RD3 containing the GSI motif shown to mediate binding to class IIa HDACs**^21^**.** The GSI motifs are highlighted in yellow and the region of NMR-defined transient helical structure in green. H, helix, E, extended. Algorithms used for secondary structure prediction were Agadir^22^ (http://agadir.crg.es/), Jpred^23^ (http://www.compbio.dundee.ac.uk/jpred4/), and Psipred^24^ (http://bioinf.cs.ucl.ac.uk/psipred/).

1293 1303 1313 1323

SDLKERTVLS GSIMQGTPRA TTESFEDGLK YPKQIKRESP

Agadir ---------- ---------- ---------- ----------

Jpred ---------- ---------- --HHHHHHHH HHHHH-----

Psipred ---HH----- ---------- ---------- ----------

**Supplementary Figure S8: Secondary structure predictions for the region of the N-CoR1 RD3 containing the GSI motif alignment-related to the one in SMRT residues 1361–1368.** Alignment was performed by Clustal Omega^25,26^ (http://toolkit.tuebingen.mpg.de/clustalw). The GSI motif is highlighted in yellow. H, helix. Algorithms used for secondary structure prediction were Agadir^22^ (http://agadir.crg.es/), Jpred^23^ (http://www.compbio.dundee.ac.uk/jpred4/), and Psipred^24^ (http://bioinf.cs.ucl.ac.uk/psipred/).

| k_a1_ (M^-1^s^-1^) | k_d1_ (s^-1^) | k_a2_ (s^-1^) | k_d2_ (s^-1^) | K_d_ (M) |
| --- | --- | --- | --- | --- |
| 1.9 × 10^3^ | 0.03 | 7.3 × 10^-3^ | 1.2 × 10^-3^ | 2.1 × 10^-6^ |

**Supplementary Table S1:** Rate constants and dissociation constant of the interaction of SMRT(1255-1452) with HDAC7 obtained from fitting SPR data.

| Protein | K_d_ (µM) |
| --- | --- |
| WT | 14 |
| R1360A | 19 |
| R1369A | - |
| V1372A | 37 |
| E1376A | 34 |
| L1379A | 7.5 |
| R1381A | 55 |
| K1384A | 43 |
| L1386A | 28 |

**Supplementary Table S2: Dissociation constants obtained by isothermal titration calorimetry for SMRT(1255-1452) alanine mutants binding to HDAC7.** K_d_ values are approximate given the levels of noise in the data and the aggregation observed at higher concentrations of HDAC7. Mutant R1369A strongly inhibits binding (see also Fig. 7A and B). Among the other mutants, R1381 shows the largest effect, a reduction in affinity by about four-fold; these effects were subsequently tested by GST pull-down assay and densitometry (Figure 7C).

References

1. Wootton, J. C. Non-globular domains in protein sequences: automated segmentation using complexity measures. *Comput. Chem.* **18,** 269–285 (1994).

2. Prilusky, J. *et al.* FoldIndex: a simple tool to predict whether a given protein sequence is intrinsically unfolded. *Bioinformatics* **21,** 3435–3438 (2005).

3. Li, X., Romero, P., Rani, M., Dunker, A. K. & Obradovic, Z. Predicting Protein Disorder for N-, C-, and Internal Regions. *Genome Inform Ser Workshop Genome Inform* **10,** 30–40 (1999).

4. Romero, P. *et al.* Sequence complexity of disordered protein. *Proteins: Structure, Function, and Bioinformatics* **42,** 38–48 (2001).

5. Obradovic, Z. *et al.* Predicting intrinsic disorder from amino acid sequence. *Proteins* **53 Suppl 6,** 566–572 (2003).

6. Linding, R. *et al.* Protein disorder prediction: implications for structural proteomics. *Structure* **11,** 1453–1459 (2003).

7. Ward, J. J., McGuffin, L. J., Bryson, K., Buxton, B. F. & Jones, D. T. The DISOPRED server for the prediction of protein disorder. *Bioinformatics* **20,** 2138–2139 (2004).

8. Dosztányi, Z., Csizmók, V., Tompa, P. & Simon, I. The pairwise energy content estimated from amino acid composition discriminates between folded and intrinsically unstructured proteins. *J. Mol. Biol.* **347,** 827–839 (2005).

9. Dosztányi, Z., Csizmok, V., Tompa, P. & Simon, I. IUPred: web server for the prediction of intrinsically unstructured regions of proteins based on estimated energy content. *Bioinformatics* **21,** 3433–3434 (2005).

10. Linding, R., Russell, R. B., Neduva, V. & Gibson, T. J. GlobPlot: Exploring protein sequences for globularity and disorder. *Nucleic Acids Res.* **31,** 3701–3708 (2003).

11. Zhang, T. *et al.* SPINE-D: accurate prediction of short and long disordered regions by a single neural-network based method. *J. Biomol. Struct. Dyn.* **29,** 799–813 (2012).

12. Walsh, I., Martin, A. J. M., Di Domenico, T. & Tosatto, S. C. E. ESpritz: accurate and fast prediction of protein disorder. *Bioinformatics* **28,** 503–509 (2012).

13. Walsh, I. *et al.* CSpritz: accurate prediction of protein disorder segments with annotation for homology, secondary structure and linear motifs. *Nucleic Acids Res.* **39,** W190-196 (2011).

14. Mészáros, B., Simon, I. & Dosztányi, Z. Prediction of protein binding regions in disordered proteins. *PLoS Comput. Biol.* **5,** e1000376 (2009).

15. Dosztányi, Z., Mészáros, B. & Simon, I. ANCHOR: web server for predicting protein binding regions in disordered proteins. *Bioinformatics* **25,** 2745–2746 (2009).

16. Codina, A. *et al.* Structural insights into the interaction and activation of histone deacetylase 3 by nuclear receptor corepressors. *Proc. Natl. Acad. Sci. U.S.A.* **102,** 6009–6014 (2005).

17. Watson, P. J., Fairall, L., Santos, G. M. & Schwabe, J. W. R. Structure of HDAC3 bound to co-repressor and inositol tetraphosphate. *Nature* **481,** 335–340 (2012).

18. Hartman, H. B., Yu, J., Alenghat, T., Ishizuka, T. & Lazar, M. A. The histone-binding code of nuclear receptor co-repressors matches the substrate specificity of histone deacetylase 3. *EMBO Rep.* **6,** 445–451 (2005).

19. Jensen, M. R., Ortega-Roldan, J.-L., Salmon, L., van Nuland, N. & Blackledge, M. Characterizing weak protein-protein complexes by NMR residual dipolar couplings. *Eur. Biophys. J.* **40,** 1371–1381 (2011).

20. Kao, H. Y., Downes, M., Ordentlich, P. & Evans, R. M. Isolation of a novel histone deacetylase reveals that class I and class II deacetylases promote SMRT-mediated repression. *Genes Dev.* **14,** 55–66 (2000).

21. Hudson, G. M., Watson, P. J., Fairall, L., Jamieson, A. G. & Schwabe, J. W. R. Insights into the Recruitment of Class IIa Histone Deacetylases (HDACs) to the SMRT/NCoR Transcriptional Repression Complex. *J. Biol. Chem.* **290,** 18237–18244 (2015).

22. Lacroix, E., Viguera, A. R. & Serrano, L. Elucidating the folding problem of alpha-helices: local motifs, long-range electrostatics, ionic-strength dependence and prediction of NMR parameters. *J. Mol. Biol.* **284,** 173–191 (1998).

23. Drozdetskiy, A., Cole, C., Procter, J. & Barton, G. J. JPred4: a protein secondary structure prediction server. *Nucl. Acids Res.* gkv332 (2015). doi:10.1093/nar/gkv332

24. Jones, D. T. Protein secondary structure prediction based on position-specific scoring matrices. *J. Mol. Biol.* **292,** 195–202 (1999).

25. Sievers, F. *et al.* Fast, scalable generation of high-quality protein multiple sequence alignments using Clustal Omega. *Mol. Syst. Biol.* **7,** 539 (2011).

26. Alva, V., Nam, S.-Z., Söding, J. & Lupas, A. N. The MPI bioinformatics Toolkit as an integrative platform for advanced protein sequence and structure analysis. *Nucleic Acids Res.* **44,** W410-415 (2016).
